# Supplementary material for: Splice-Junction-Based Mapping of Alternative Isoforms in the Human Proteome
Source: Cell Rep. Author manuscript; Available in PMC 2020 Jan 15. (PMC6961840; doi:10.1016/j.celrep.2019.11.026)
Supplement: 2 [file NIHMS1546469-supplement-2.zip › DF1/PXD010154/Heart/Heart_48_CLTCL1_LQEHFQLQNLGINPANIGFSTLTMESDK.pdf]

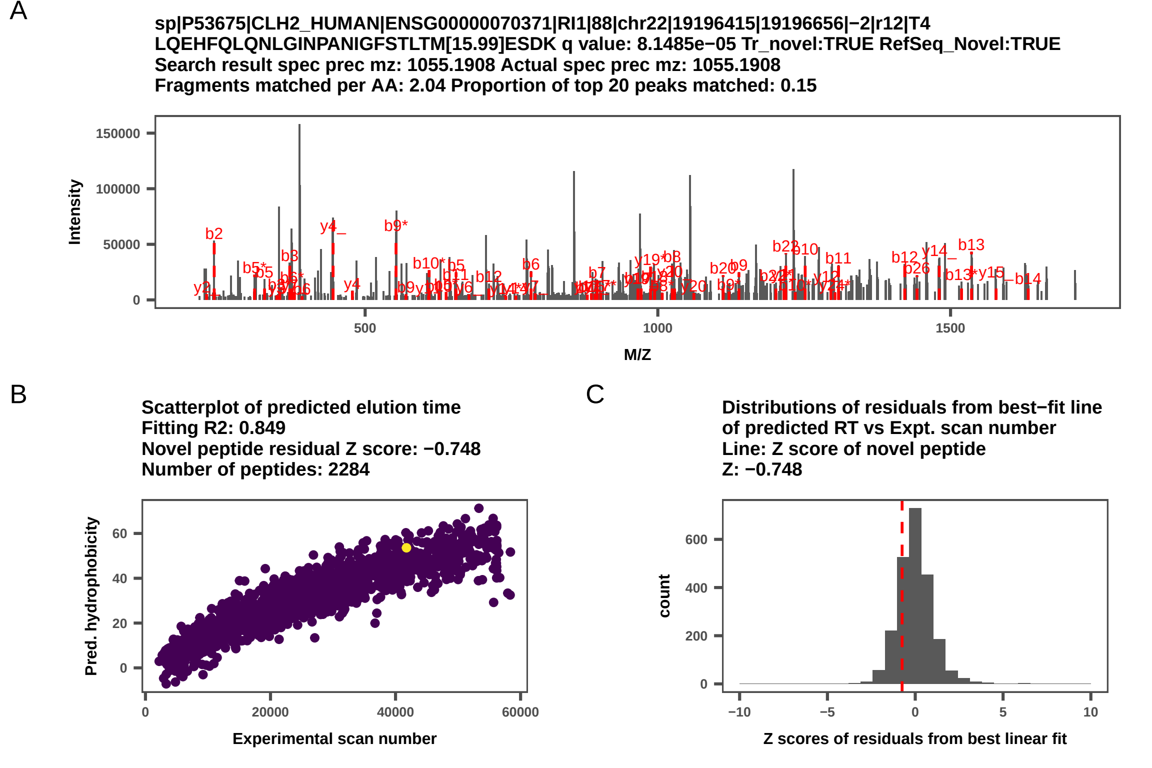
A
sp|P53675|CLH2_HUMAN|ENSG00000070371|RI1|88|chr22|19196415|19196656|−2|r12|T4
LQEHFQLQNLGINPANIGFSTLTM[15.99]ESDK q value: 8.1485e−05 Tr_novel:TRUE RefSeq_Novel:TRUE
Search result spec prec mz: 1055.1908 Actual spec prec mz: 1055.1908
Fragments matched per AA: 2.04 Proportion of top 20 peaks matched: 0.15
Intensity
150000
100000
50000
0
b2
b3
y4_
b9*
b10*
b5
b6
b11
b12
b7
b13
y14_
b26
b10
b20
b9
y4
y2
y20
M/Z
500
1000
1500
B
Scatterplot of predicted elution time
Fitting R2: 0.849
Novel peptide residual Z score: −0.748
Number of peptides: 2284
Pred. hydrophobicity
60
40
20
0
0
20000
40000
60000
Experimental scan number
C
Distributions of residuals from best−fit line
of predicted RT vs Expt. scan number
Line: Z score of novel peptide
Z: −0.748
count
600
400
200
0
−10
−5
0
5
10
Z scores of residuals from best linear fit
